# The Importance of the Interaction of CheD with CheC and the Chemoreceptors Compared to Its Enzymatic Activity during Chemotaxis in *Bacillus subtilis*

**DOI:** 10.1371/journal.pone.0050689

**Published:** 2012-12-03

**Authors:** Wei Yuan, George D. Glekas, George M. Allen, Hanna E. Walukiewicz, Christopher V. Rao, George W. Ordal

**Affiliations:** 1 Department of Biochemistry, University of Illinois at Urbana-Champaign, Urbana, Illinois, United States of America; 2 Department of Chemical and Biomolecular Engineering, University of Illinois at Urbana-Champaign, Urbana, Illinois, United States of America; Centre National de la Recherche Scientifique, Aix-Marseille Université, France

## Abstract

*Bacillus subtilis* use three systems for adaptation during chemotaxis. One of these systems involves two interacting proteins, CheC and CheD. CheD binds to the receptors and increases their ability to activate the CheA kinase. CheD also binds CheC, and the strength of this interaction is increased by phosphorylated CheY. CheC is believed to control the binding of CheD to the receptors in response to the levels of phosphorylated CheY. In addition to their role in adaptation, CheC and CheD also have separate enzymatic functions. CheC is a CheY phosphatase and CheD is a receptor deamidase. Previously, we demonstrated that CheC’s phosphatase activity plays a minor role in chemotaxis whereas its ability to bind CheD plays a major one. In the present study, we demonstrate that CheD’s deamidase activity also plays a minor role in chemotaxis whereas its ability to bind CheC plays a major one. In addition, we quantified the interaction between CheC and CheD using surface plasmon resonance. These results suggest that the most important features of CheC and CheD are not their enzymatic activities but rather their roles in adaptation.

## Introduction

During the process of chemotaxis, *Bacillus subtilis* travels towards higher concentrations of attractants, such as amino acids, sugars and oxygen [1], [2], [3], or towards lower concentrations of repellents, such as pentachlorophenol and indole [4], [5]. One important aspect of chemotaxis is adaptation, where cells respond only transiently to changes in attractant or repellent concentrations. For example, the addition of attractant greatly increases smooth swimming in cells. However, the cells eventually return to their prestimulus random behavior, characterized by alternate periods of smooth swimming and reorientating tumbles [6], [7]. The transient increase in smooth swimming due to the addition of attractant is caused by a transient increase in CheA activity, a histidine kinase that associates with the receptors and a coupling protein, CheW. The phosphoryl group from CheAp is then transferred to CheY, a response regulator that binds to the flagellar motors and induces smooth swimming [8]. Note, this process is different in *Escherichia coli* where attractants decrease CheA activity and CheYp induces tumbles.

*B. subtilis* has three systems that foster adaptation: a system involving receptor methylation [5], [9], a system involving phosphorylation of CheV [10], [11], and a system involving CheC, CheD, and CheYp [12], [13]. CheV is homologous to the coupling protein CheW except that it also has an N-terminal response regulator domain [14], [15]. CheC and CheD are proteins not found in *E. coli* but are widespread among nearly all groups of bacteria and are known to interact with one another [16], [17]. Note, *E. coli* has only a single adaptation system: it uses receptor methylation [18].

The CheC/CheD/CheYp adaptation system is the focus of this work. The cornerstone of this system is CheD. This protein is believed to activate the CheA kinase by binding to the receptors. CheD’s ability to bind the receptors is controlled by CheC through a competitive binding mechanism. Moreover, CheYp increases the affinity of CheD for CheC. The essence of this adaptation mechanism is that CheYp controls CheD binding to the receptors through its interactions with CheC. When CheYp levels are high, CheC is a better binding target for CheD than the receptors. The result is that CheA activity is decreased as less CheD is bound to the receptors [12], [13], [19].

One of the paradoxes in the study of chemotaxis in *B. subtilis* is that CheC and CheD both have enzymatic activities; however, these enzymatic activities are not required for the CheC/CheD/CheYp adaptation system. CheC is a CheYp phosphatase and its activity is five-fold higher in presence of CheD. However, CheC activity is only 6% that of a protein of likely common evolutionary origin, the switch protein FliY, which is the main CheYp phosphatase [16]. Mutating residues in CheC that abolish binding to CheD have a far more deleterious effect on chemotaxis than mutating residues that abolish CheYp phosphatase activity [12], [20]. This finding was supported by the properties of one particular mutant in *cheC* in which aspartate-149, which lies at the CheD binding interface, is converted to a lysine [21]. *CheC-D149K* showed very poor chemotaxis on swarm plates, similar to a *cheC* null mutant. CheC-D149K also showed considerably reduced binding to CheD in pulldown experiments. However, CheC-D149K had normal CheYp phosphatase activity.

CheD is a deamidase for particular glutamine residues in receptors [21], [22]. Mutants in *cheD* activate CheA kinase very poorly and upon addition of attractant show diminished activation. Their receptors are also very poorly methylated. These results imply that without CheD the receptors adopt a very refractory phenotype [19], [23]. Interestingly, the binding site for CheD on the receptors where deamidation is catalyzed is very similar to the binding site on CheC; in fact, a glutamine can be engineered in CheC in place of a glutamate in the correct location and CheD will deamidate that glutamine [21]. We hypothesize that the main purpose of CheD during chemotaxis is to bind to receptors and CheC as part of the CheC/CheD/CheYp adaptation system and not to deamidate receptors. In the present study, we present data in support of this hypothesis.

## Results

### Effect of CheYp on the Affinity for CheC for CheD

Our working model of the CheC/CheD/CheYp adaptation system assumes that CheYp controls the strength of the interaction between CheC and CheD. To test this hypothesis, we employed surface plasmon resonance (SPR). The virtue of this technique is that both the k_on_ and k_off_ rates can be estimated and the dissociation constant K_D_ can be calculated from their quotient (k_off_/k_on_). In these experiments, GST-6xHis-CheD was immobilized on an nitrilotriacetic acid (NTA) chip and different concentrations of CheC were flowed by and allowed to associate with CheD for four minutes, followed by two minutes of buffer to allow dissociation. By including CheY with CheC, with or without an excess of acetyl phosphate to convert CheY to CheYp, we assessed changes in the interaction between CheD and CheC. Whereas presence of CheY caused an almost two-fold decrease in the affinity of CheD for CheC, the presence of CheYp caused a more than seven-fold increase in affinity (**Table 1**). Thus, by converting CheY to CheYp, there was a large increase in affinity of the associated CheC for CheD. Virtually all of this increased affinity was due to an increased “k_on_” rate.

**Table 1:** Kinetic parameters of CheC-CheD binding from SPR experiments.

	k _off_		k _on_	K _D_
Unit	S^−1^		M^−1^S^−1^	M
CheC-CheD	0.0568+/−0.0089	14733+/−2476		3.95E-06+/−1.06E-06
CheC-CheD-CheY	0.0743+/−0.0079	10627+/−1184		7.01E-06+/−5.30E-07
CheC-CheD-CheYp	0.0976+/−0.0057	178967+/−13284		5.46E-07+/−3.31E-08

### Separating the Effects of CheD Binding and Deamidase Activity

**Figure 1:**
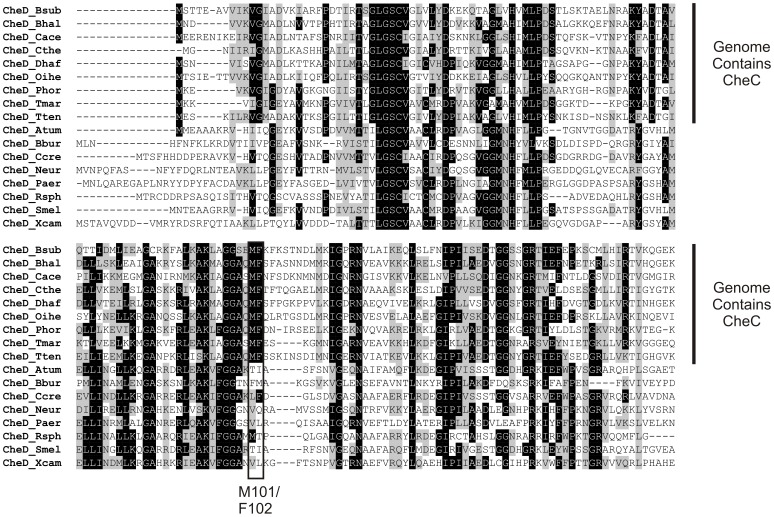
Sequence alignment of various CheDs shows that residues M101 and F102 (boxed) are conserved in species that also contain CheC. Abbreviations for species that contain CheC: Bsub, *Bacillus subtilis*; Bhal, *Bacillus halodurans*; Cace, *Clostridium acetobutylicum*; Cthe, *Clostridium thermocellum*; Dhaf, Desulfitobacterium hafniense; Oihe, Oceanobacillus iheyensis; Phor, *Pyrococcus horikoshii*; Tmar, *Thermotoga maritima*; Tten, *Thermoanaerobacter tengcongensis*. Abbreviations for species that do not contain CheC: Atum, *Agrobacterium tumefaciens*; Bbur, Borrelia burgdorferi; Ccre, Caulobacter crescentus; Neur, *Nitrosomonas europaea*; Paer, *Pseudomonas aeruginosa*; Rsph, *Rhodobacter sphaeroides*; Smel, *Sinorhizobium meliloti*; Xcam, *Xanthomonas campestris*.

**Figure 2:**
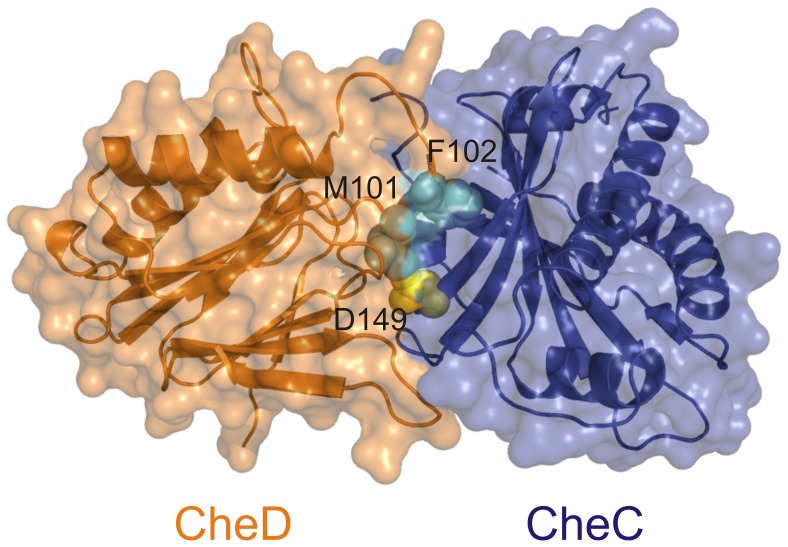
A CheC/CheD structural model based on the *T. maritima* crystal structure (PDB ID 2F9Z) shows residues at the binding interface [**21**]. Residues M101 and F102 on CheD are highlighted in cyan, and residue D149 on CheC is highlighted in yellow.

Our governing hypothesis is that CheD functions primarily as a regulator of CheA activity as opposed to as a receptor deamidase. To test this hypothesis, we sought to isolate *cheD* mutants that were enzymatically active but were unable to support chemotaxis. To identify such a mutant, we surveyed bacterial genomes to discover conserved residues in CheD present in species having CheC but not in species lacking CheC (**Figure 1**). Such residues are likely important for the binding of CheD to CheC and possibly the receptors. We then used the crystal structure of CheC/CheD from *Thermatoga maritima*, threaded onto it the sequence of *B. subtilis* CheC and CheD, and looked for residues at the interface. Two residues stood out as possibilities, methionine-101 and phenylalanine-102, since they were always conserved in species possessing CheC but not in those lacking CheC. Methionine-101 sits at the interface between the two proteins and very likely fosters hydrophobic interaction between the two proteins; phenylalanine-102 intercalates within the neighboring α-helix in CheC (**Figure 2**).

**Figure 3:**
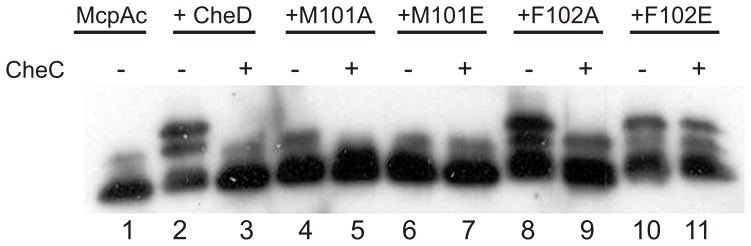
Western blots of *in vitro* deamidation assays of McpAc show CheD-mediated receptor modifications. Deamidation of the receptor fragment results in higher-migrating bands on a polyacrylamide gel. CheC is added, as indicated, and inhibits deamidation. Lanes 2 and 3 show McpAc incubated with wild-type GST-CheD, and lanes 4–11 show McpAc incubated with various GST-CheD mutants.

Both CheD-M101A and CheD-M101E showed little deamidase activity on McpA, whereas CheD-F102A as well as CheD-F102E showed approximately normal deamidase activity (**Figure 3**). In this assay, the C-terminal fragment of McpA (McpAc) was incubated in the presence or absence of CheD and the resulting mixture was separated by SDS-PAGE, followed by Western analysis using anti-McpA antibody. Due to the negative charge on the deamidated receptors, which thus bind less SDS, the deamidated receptors migrate more slowly than the unmodified receptors [22]. Unlike most other receptors such as McpC, McpA and McpAc show a large shift upon changes in degree of amidation or methylation at the pertinent sites when measured using SDS-PAGE [22]. CheC interfered fairly well with CheD-F102A but only slightly with CheD-F102E, probably reflecting poorer binding to the latter. Based on these results CheD-F102E was selected for further study as we wished to select a mutant having deamidase activity but poor chemotaxis due to reduced binding of the mutant CheD to CheC.

### Confirmation of the CheD/CheC Interaction by a Pulldown Experiment

**Figure 4:**
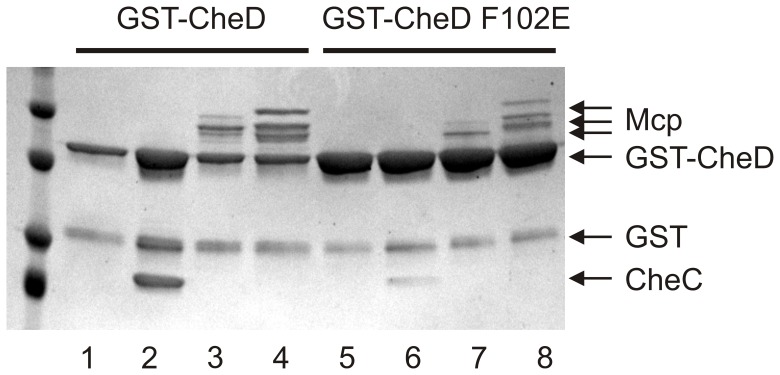
GST pulldowns with CheD’s two binding partners (CheC and McpAc) show reduced binding by GST-CheD-F102E. Lanes 1 (GST-CheD only control), 2 (+CheC), 3 (+McpAc) and 4 (+McpCc) contain GST-CheD bound to glutathione beads. Lanes 5 (GST-CheD-F102E only control), 6 (+CheC), 7 (+McpAc) and 8 (+McpCc) contains GST-CheD-F102E bound to glutathione beads. Multiple bands of the Mcps correspond to receptor deamidation.

Diminished binding of mutant CheD-F102E both to CheC and to receptors was confirmed in a pulldown experiment. In this experiment glutathione-S-transferase-CheD (GST-CheD) or GST-CheD-F102E was bound to glutathione-beads, then incubated with CheC, McpAc or McpCc and washed. The bound GST-CheD was eluted with glutathione. The eluents were separated by SDS-PAGE and the gels were stained. GST-CheD bound CheC more tightly than GST-CheD-F102E (**Figure 4** **, lanes 2 and 6**). Image analysis revealed that GST-CheD-F102E binds roughly 10% of the CheC when compared to GST-CheD. Similarly, GST-CheD bound McpAc and McpCc more tightly than GST-CheD-F102E (**Figure 4** **, lanes 3, 4 versus 7, 8**), with the mutant CheD binding roughly a third of the receptor compared to GST-CheD. We note that during the course of incubation both GST-CheD and GST-CheD-F102E catalyzed deamidation of McpAc and McpCc to produce multiple bands on the gel (**Figure 4** **, lanes 3,4,7 and 8**). We also used SPR to analyze the interaction between CheD-F102E and CheC. However, no binding was observed, likely because the interaction is too weak to detect with the technique.

### Effect on Chemotaxis of Mutations in CheC and in CheD Affecting the Affinity of CheC and CheD

**Figure 5:**
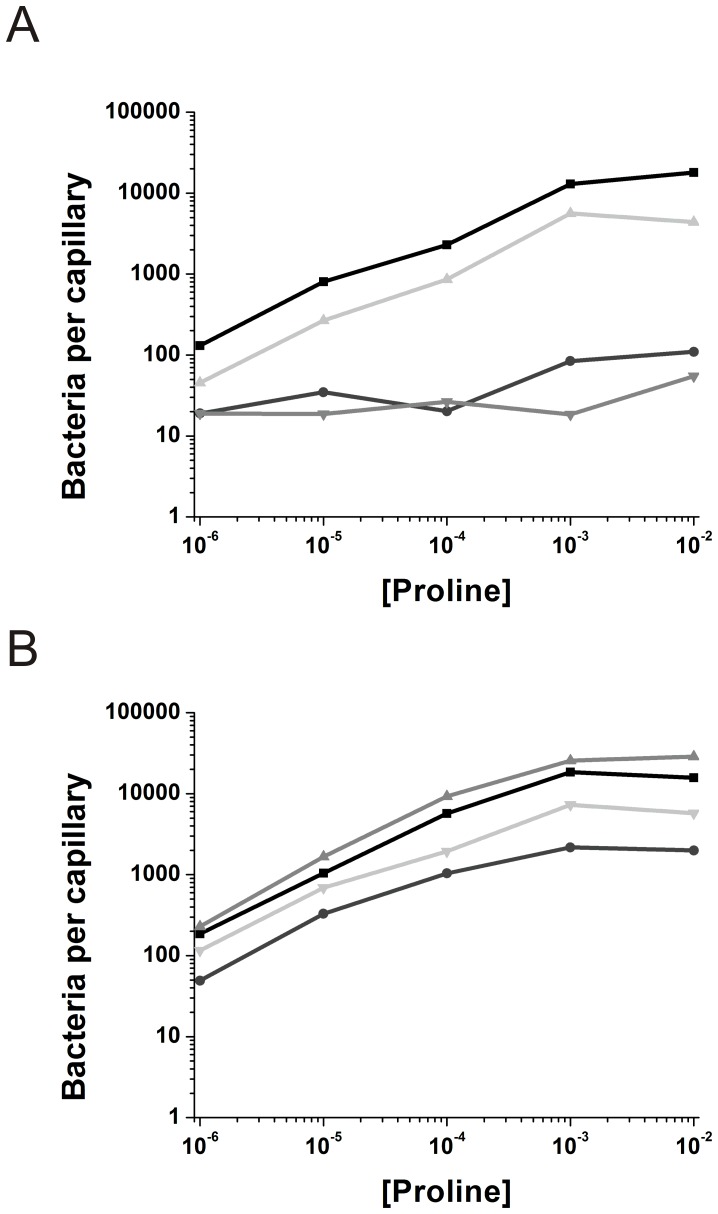
Capillary assays show reduced chemotactic ability in various mutant strains. A. Wild-type OI1085 (▪), *cheD* knockout OI2934 (•), *cheD*+pEB112::*cheD* OI3142 (▴) and *cheD*+pEB112::*cheD (F102E)* OI4488 (▾). B. Wild-type OI1085 (▪), *cheC* knockout OI3135 (•), *cheC amyE::cheC* OI3165 (▴) and *cheC amyE::cheC (D149K)* OI4172 (▾). 1 mM IPTG was added to all the complementation strains.

The identity of attractants or repellents whose chemotaxis is mediated via McpA are unknown. In order to determine effects of mutations in CheD on chemotaxis, we used McpC, which is the sole receptor for proline taxis. *CheC-D149K* was previously found to show poor chemotaxis on swarm plates and exhibit reduced affinity for CheD [21]. We sought to confirm and extend this analysis using the capillary assay. The *cheC* mutant (*cheC-D149K*) was less tactic toward proline than the wild type but more than the *cheC* null mutant (**Figure 5B**). Using this same methodology, but with a mutant *cheD*, namely *cheD-F102E*, we found that proline taxis was reduced to the same extent as in the *cheD* null mutant (**Figure 5A**). Receptor deamidation, which is normal for the CheD-F102E deamidase (**Figure 3**), does not seem to be important for chemotaxis. As expected, restoring CheD using a plasmid having *cheD* (**Figure 5A**) or restoring CheC using a plasmid having *cheC* (**Figure 5B**) largely restored chemotaxis. The restoration was not complete in the case of CheD. This degree of reduction would be expected if in this strain *cheD* was somewhat polar on *cheC*, which is immediately downstream in the genome [24]. Thus, good chemotaxis depends on the mutual affinity of CheC and CheD and impairing this interaction either by a mutation in CheC or CheD impairs chemotaxis.

## Discussion

The enzymatic activity of a protein is often assumed to determine its function in a biological process. However, in the case of CheC, a CheYp phosphatase, and CheD, a receptor deamidase, this assumption does not appear to be true. In the case of CheC, the mutant *cheC-D149K* showed similar chemotactic ability as the *cheC* null mutant (**Figure 5**, [21]). However, CheC-D149K dephosphorylated CheYp normally but bound poorly to CheD [21]. In the case of CheD, the mutant *cheD-F102E* showed similar chemotaxis ability as the *cheD* null mutant (**Figure 5**). However, CheD-F102D deamidated McpA normally (**Figures 3** **and** **4**) but bound CheC and McpAc with lower affinity than CheD (**Figure 4**). The reason, of course, for the importance of the binding functions of CheC and CheD compared to their enzymatic functions appears to be that CheC and CheD participate in adaptation.

The SPR results confirm the prediction from the hypothesis that the affinity of CheD for CheC is increased when the levels of CheYp increase, an event that occurs, via an increase in CheAp levels, immediately after addition of attractant [8]. What was remarkable to us was that this increase in affinity was almost entirely due to an increase in the k_on_ rate (**Table 1**). Thus, it would appear that an event that occurs very shortly after CheYp levels increase is that CheYp binds to CheC and CheC quickly binds CheD, presumably to reduce the free CheD concentration in the cytoplasm. CheD would then dissociate from the receptors. The great increase in the k_on_ rate would facilitate this process. We do not know the rate of dissociation of CheD from the receptors nor whether it might be increased by binding attractant to the receptors. In future work, we hope to investigate the rate of dissociation of CheD from receptors, both in the attractant-bound and attractant-free state, in order to get further insight into the working of the CheC/CheD/CheYp adaptation system.

## Materials and Methods

### Strains and Plasmids

All of the strains and plasmids used in this study are listed in **Table 2**. All of the *B. subtilis* strains are derived from the chemotactic strain (Che*+*) OI1085. All cloning and plasmid propagation was performed in *E. coli* strains TG1. Recombinant proteins were overexpressed in *E. coli* strains RP3098 and BL-21. Plasmids used for protein purification were expressed using either pUSH1 or pGEX-6P-2.

**Table 2:** Strains and plasmids used in this study.

Strain or Plasmid	Relevant genotype or description	Reference
OI1085	Che+, *trpF7 hisH2 metC133*	[27]
OI2934	*cheD1::cat*	[23]
OI3135	*ΔcheC1*	[23]
OI3142	*cheD1::cat* pEB112::*cheD*	[23]
OI3165	*ΔcheC1 amyE5720::cheC*	[23]
OI4172	*ΔcheC1 amyE5720::cheC (D149K)*	[21]
OI4488	*cheD1::cat* pEB112::*cheD (F102E)*	This work
TG-1	*E. coli* cloning host	Amersham
BL-21	*E. coli* protease deficient expression host	Amersham
RP3098	*E. coli* Δ(*flhD-flhB*)4, *che*	J.S.Parkinson
pEB112	*B. subtilis-E. coli* shuttle vector	[23]
pGEX-6P-2	GST-Tag Expression plasmid (Amp^R^)	Amersham
pUSH1	*B. subtilis-E. coli* shuttle vector for His-tag fusions	[28]
pAIN620	pUSH1 expressing 6xHis-McpA cytoplasmic domain	[22]
pHS102	pGEX-6P-2::*cheY1*	[25]
pTM18	pGEX-6P-2::*cheC2*	[16]
pTM25	pGEX-6P-2::*cheD2*	[16]
pWY79	pGEX-6P-2::*cheD2 (M101A)*	This work
pWY80	pGEX-6P-2::*cheD2 (M101E)*	This work
pWY81	pGEX-6P-2::*cheD2 (F102A)*	This work
pWY82	pGEX-6P-2::*cheD2 (F102E)*	This work
pWY88	pGEX-6P-2::*6xHis-cheD2*	This work

The GST-fusion pGEX-6P-2 plasmids were made by amplifying the *cheD* gene from OI1085 genomic DNA with PCR using primers that incorporated EcoRI and NotI restriction sites. This fragment was then ligated into pGEX-6P-2. Subsequent point mutations on this plasmid were made using Quickchange (Stratagene) mutagenesis.

Strain OI4488 was constructed by mutating the pWN5 (pEB112::*cheD*) plasmid using Quickchange mutagenesis and transforming it into OI2934, selecting for kanamycin resistance [23]. Expression of CheD-F102E was confirmed using western blots (data not shown) and found to be at wild-type levels when induced with 1 mM isopropyl β-D-1-thiogalactopyranoside (IPTG).

### Protein Purification

The chemoreceptor carboxy-terminal fragment of McpA, which extends from the second transmembrane spanning region to the C-terminus of the protein (McpAc), was purified as a 6xHis-fusion under denaturing condition as previously described [22]. A 2-liter culture of LB (1% (w/v) tryptone, 0.5% (w/v) yeast extract, 0.5% (w/v) NaCl) plus 10 µg/ml chloramphenicol was inoculated 1∶100 with an overnight culture of RP3098 harboring pAIN620 and grown at 37°C with agitation (250 rpm) for 9 hours. The cells were harvested by centrifugation at 5000×g for 5 min, resuspended in Buffer B (8 M urea, 0.1 M NaH_2_PO_4_, 0.01 M Tris, pH 8) and incubated at room temperature for 1 h with mixing. The supernatants were clarified by two serial centrifugations (7000×g, 5 min; 40,000×*g*, 40 min) and loaded onto 5 mL Hi-Trap Chelating column charged with 0.1 M NiSO_4_ paired to an AKTA Prime FPLC system (GE Healthcare). The column was washed with 10 volumes Buffer B, followed by 10 washes with Buffer C (Buffer B at pH 6.3). Elution was performed with 25 mL Buffer E (Buffer B at pH 4.5). These samples were dialyzed against three changes of 25 mM NH_4_HCO_3_ at 4°C, and aliquots were frozen at −80°C.

The GST-fusion proteins were purified as previously described [25]. A stationary phase overnight culture was diluted 1∶100 into 6 liters of LB with 100 µg/ml ampicillin and grown at 37°C (250 rpm) to an A_600_ of 0.6. IPTG was then added to a final concentration of 1 mM. The GST-CheC, GST-CheD and GST-6xHis-CheD cultures were incubated at 250 rpm overnight at room temperature, and the GST-CheY culture was incubated at 15°C (200 rpm) for 48 hours. Cells were spun down at 8,000×g for 8 min and resuspended in TBS (50 mM Tris, pH 7.5, 150 mM NaCl) buffer followed by sonication. The supernatants were clarified by two rounds of centrifugations (9,000×g, 15 min; 40,000×g, 40 min), and applied to 5-ml GSTrap columns pre-washed with 10 column volumes of TBS buffer. Protein bound columns were then washed with at least 15 column volumes of TBS buffer, and GST tagged proteins was eluted with using 10 ml of glutathione elution buffer (GEB; 50 mM Tris, 5 mM glutathione, pH 8). To remove the GST tag, the purified GST-CheC or GST-CheY was cleaved by PreScission protease, as specified by the supplier (Amersham Biosciences), and applied to another 5 ml GSTrap column. The flow-through was collected and concentrated to ∼5 ml using a cellulose ultrafiltration membrane (Millipore) in an Amicon ultrafiltration cell. Then the purified proteins were dialyzed in TKMD buffer (50 mM Tris, pH 8.0, 50 mM KCl, 5 mM MgCl_2_, 0.1 mM dithiothreitol (DTT)) and aliquots were stored at −80°C.

### Surface Plasmon Resonance (SPR)

SPR analysis was performed using the Biacore 3000 system and NTA sensorchips (GE Health). The sensorchip was activated according to the manufacturer’s instructions, and the buffers used in the microfluidic system were as follows: running buffer (TKMD), regeneration buffer (0.35 M EDTA in running buffer), and nickel solution (0.5 M NiCl_2_ in running buffer). A flow rate of 20 µl/min was used for all the experiments.

The chip was pre-cleaned by injection of 20 µl regeneration buffer, and then charged with 20 µl of nickel solution. 20 µl of 0.1% BSA was then injected to remove background binding. Then 500 response units (RU) of GST-6xHis-CheD was immobilized on three different flow cells (2, 3, and 4), while the flow cell 1 was used as a mock immobilized, blank control. After immobilization, 80 µl of CheC at different concentrations was injected, respectively, to study CheD-CheC binding kinetics.

To study CheD-CheC-CheY binding kinetics, different from the above, 20 µM of CheY in TKMD was used as the running buffer throughout the experiment. During injection of CheC, the same concentration of CheY was also added. Similarly, to study CheD-CheC-CheYp binding kinetics, 5 mM of acetyl phosphate (a small-molecule phospho-donor) and 20 µM of CheY in TKMD was used as the running buffer [12]. The same concentrations of Ac-P and CheY were also added while injecting CheC.

After each round of binding and dissociation, the NTA chip was regenerated and charged again for the next round of study. The SPR curves were then processed and analyzed using BIAevaluation software as per the manufacturer’s specifications (GE Healthcare). The experiments were run in triplicate on separate days to ensure reproducibility.

### Western Blot

Western blots were performed as previously described [26]. Samples were separated by SDS-PAGE and then transferred to PDVF membrane (Millipore) by semi-dry transfer, using transfer buffer (50 mM Tris, 40 mM glycine, 0.15 mM SDS, 20% (v/v) methanol). The membrane was blocked for 4 h with blocking buffer (20 mM Tris, pH 7.5, 250 mM NaCl, 5% (w/v) milk powder, 0.05% Tween 20), then the anti-McpA antibody was added for overnight incubation at a dilution of 1∶20,000. The membranes were then washed and incubated with a 1∶20,000 dilution of the secondary antibody (HRP-conjugated goat-anti-rabbit-IgG, Pierce) in blocking buffer for 2 h. Membranes were washed and then treated with ECL Plus signal solution per the manufacturer’s specifications (GE Healthcare). Proteins were visualized using a UVP EpiChem^3^ Darkroom and LabWorks Image Acquisition and Analysis Software.

### Receptor Deamidation Assay

Deamidation reactions were performed as previously described [22]. Briefly, 1 µmol of McpAc was incubated for 1 h with equal concentrations of CheD or mutated CheD at room temperature in reaction buffer (50 mM Tris, pH 7.5, 0.1 mM dithiothreitol, 1 mM MgCl_2_, 0.5 mM EDTA). Samples (10 µl) were then mixed with 10 µl of 2×SDS loading buffer to stop the reaction. Immunoblotting was performed as described above. To investigate inhibition caused by CheC, 2 µmol of CheC and 2 µmol of CheD were used in the incubation of McpAc.

### GST Pulldowns

The pulldowns were performed as previously described [21]. 100 µl of 80 µM GST-CheD or GST-CheD-F102E was added to 50 µl of pre-washed (with 400 µl TBS-X (TBS +1% Triton X-100)) glutathione beads (GE Healthcare) in a Handee spin cup column (Pierce) and incubated for 10 min. The beads were washed with 800 µl TBS-X, and 100 µl of 100 µM of receptor cytoplasmic fragment (McpAc) or CheC was added and incubated for 10 minutes. The beads were again washed with 800 µl TBS-X, and the protein was eluted with 75 µl GEB. 25 µl of 4X SDS solubilizer was added and the samples were run on SDS-PAGE gels and visualized with Coomassie stain. A GST control was also run, but no binding of CheC or McpAc was seen (data not shown). Densitometry image analysis was performed using Labworks Image Analysis Software (UVP Bioimaging Systems).

### Capillary Assay for Chemotaxis

The capillary assay was performed as described by Zimmer et al (2002). Briefly, cells were grown overnight on tryptose blood agar plates, suspended in a minimal medium, grown about six hours, and put into a suitable capillary assay medium. Cells were introduced into a “pond” into which a capillary containing attractant was then inserted. Cells entering the capillary were harvested after half an hour, extruded into broth, and plated on suitable rich medium plates. Colonies were counted the next day. Each experiment was performed in triplicate on at least two separate days to ensure reproducibility.
